# Truth Telling and Treatment Strategies in End-of-Life Care in Physician-Led Accountable Care Organizations: Discrepancies Between Patients’ Preferences and Physicians’ Perceptions

**DOI:** 10.1097/MD.0000000000000657

**Published:** 2015-04-24

**Authors:** Hsien-Liang Huang, Shao-Yi Cheng, Chien-An Yao, Wen-Yu Hu, Ching-Yu Chen, Tai-Yuan Chiu

**Affiliations:** From the Department of Family Medicine (HLH), Cardinal Tien Hospital, and Fu-Jen Catholic University, New Taipei City; Department of Family Medicine (SYC, CAY, CYC, TYC); and School of Nursing (WYH), College of Medicine and Hospital, National Taiwan University, Taipei, Taiwan.

## Abstract

Providing patient-centered care from preventive medicine to end-of-life care in order to improve care quality and reduce medical cost is important for accountable care. Physicians in the accountable care organizations (ACOs) are suitable for participating in supportive end-of-life care especially when facing issues in truth telling and treatment strategy. This study aimed to investigate patients’ attitudes toward truth telling and treatment preferences in end-of-life care and compare patients’ attitudes with their ACOs physicians’ perceptions.

This nationwide study applied snowball sampling to survey physicians in physician-led ACOs and their contracted patients by questionnaire from August 2010 to July 2011 in Taiwan. The main outcome measures were beliefs about palliative care, attitudes toward truth telling, and treatment preferences.

The data of 314 patients (effective response rate = 88.7%) and 177 physicians (88.5%) were analyzed. Regarding truth telling about disease prognosis, 94.3% of patients preferred to be fully informed, whereas only 80% of their physicians had that perception (*P* < 0.001). Significant differences were also found in attitudes toward truth telling even when encountering terminal disease status (98.1% vs 85.3%). Regarding treatment preferences in terminal illness, nearly 90% of patients preferred supportive care, but only 15.8% of physicians reported that their patients had this preference (*P* < 0.001).

Significant discrepancies exist between patients’ preferences and physicians’ perceptions toward truth telling and treatment strategies in end-of-life care. It is important to enhance physician–patient communication about end-of-life care preferences in order to achieve the goal of ACOs. Continuing education on communication about end-of-life care during physicians’ professional development would be helpful in the reform strategies of establishing accountable care around the world.

## INTRODUCTION

Providing patient-centered care from preventive medicine to end-of-life care in order to improve care quality and reduce medical cost is important for accountable care.^1–3^ However, with much expansion of life-sustaining advanced technologies, end-of-life care may be filled with burdensome interventions of little clinical value.^4,5^ In such circumstances, the role of accountable care organizations (ACOs) as continuing and coordinative care of patients makes physicians in the ACOs suitable for participating in supportive end-of-life care especially when facing issues in truth telling and treatment strategy.^6–9^

Protecting patient autonomy to ensure that their treatment preferences are supported is an essential responsibility for physicians in ACOs during comprehensive, coordinated, and continuing care.^10,11^ Patient values should be respected during informed consent processes, and their preferences should be implemented in all medical decision making. The decision-making process requires sufficient patient understanding of the relevant diagnostic information, but truth telling by physicians is a frequently encountered ethical dilemma.^12–14^ Physicians in ACOs who are acquainted with the patient and family should better understand the values and emotional reactions of the patient, and they could involve better in promoting patients using advance directives to choose preferred treatment preference.^15–18^

Previous studies mostly focus on health professionals and patients in secondary or tertiary care setting, and differences in attitudes toward certain medical decisions have been observed in previous studies.^19–21^ Little is known on patients and their physicians in ACOs, and few surveys have investigated the perceptions of physicians compared with the preferences of their contracted patients. Surveying patients’ preferences toward truth telling and treatment strategies may provide important information for physicians and policy makers in establishing a better end-of-life care network, and this kind of survey should be adopted in other countries such as United States after the enactment of Patient Protection and Affordable Care Act due to the quality measurement of ACOs by patients’ experiences may be difficult in end-of-life care.^10^ Moreover, community-based physician-led ACOs could provide better end-of-life care as patients’ wishes on limiting unnecessary image or blood testing, invasive procedures, and hospitalizations.^5,22^ To understand patient treatment preference and respect their wishes in end-of-life care, the study aimed to investigate the attitude toward truth telling and treatment preference of patients and identify the perception of their physicians in community-based physician-led ACOs.

## METHODS

### Design and Participants

This study is a nationwide survey using the snowball sampling method to survey physicians in community-based physician-led ACOs and their contracted patients using a structured, validated questionnaire. The questionnaire was mailed to physicians of the community medical teams (CMTs) in Taiwan from August 2010 to July 2011. Physicians in the CMTs represent different specialties and are certified to conduct family practice in the community after a set of training courses. Since 2003, as part of Taiwan's National Health Insurance System, the CMTs have functioned as physician-led ACOs and offer patient-centered integrated care, including preventive medicine, health promotion, 24-hour on-call service, effective referral system, and end-of-life care. Return and completion of the questionnaire represented consent to participate. The questionnaire was resent to those who did not return the reply postcard within 2 weeks after the initial mailing. The design of the study and participants’ selection were approved by the ethics committee of the National Taiwan University Hospital and the National Science Council of Taiwan.

### Questionnaire

A structured, self-reported questionnaire consisting of 5 parts was administered to all participants. The 5 parts included questions on demographic characteristics, beliefs about palliative care, subjective norms, attitudes toward truth telling, and attitudes toward treatment preferences. The entire questionnaire was tested for content validity by a panel comprising 7 experts in end-of-life care. Each item in the questionnaire was scored on a 5-point scale ranging from “very inappropriate and not relevant” (1) to “very appropriate and relevant” (5). Only the items that had an average rating above “4” were included in the final questionnaire. Additionally, 15 physicians and members of the general public completed the questionnaire to confirm its face validity and ease of application.

Demographic characteristics assessed by the questionnaire included sex, age, marital status, education level, religion, and past experience in palliative care. The remaining 4 parts included the following.

#### Beliefs About End-of-Life Care

This part was designed with careful scrutiny of the literature by the investigators and included respondents’ perceptions about the threats, benefits, and barriers of hospice care. The measure is a 26-item set in the physician questionnaire and a 24-item set in the patients’ questionnaire, and both use a 5-point Likert scale ranging from “strongly disagree” (1) to “strongly agree” (5). Bartlett test (BT) of sphericity and the Kaiser–Meyer–Olkin (KMO) test were used to determine that beliefs data were suitable for exploratory factor analysis. The BT values for the data were 2568 for physicians and 5744 for patients. Both significant values were 0.000. The KMO value was 0.851 for physicians and 0.891 for patients. The draft items were analyzed using principal component factor analysis followed by orthogonal varimax rotation. The beliefs measure was constructed using threats, benefits of quality-of-life care and good death, and barriers perceived. Internal consistency was demonstrated with the Cronbach α coefficient range of 0.56–0.934 in the subscales of beliefs.

#### Subjective Norms

This part is defined as “the motivation to comply with significant others’ opinions” to provide or receive hospice care. The measure has 6 items, the influences of spouses, sons, daughters, colleagues, friends, or others, and used a 5-point Likert scale ranging from “strongly unaffected” (1) to “strongly affected” (5).

#### Truth Telling

This section identifies patients’ attitudes toward truth telling and physicians’ perceptions about respecting patients’ choices. The need for truth telling was assessed under 3 conditions of health status: overall disease prognosis, cancer diagnosis, and terminal status.

#### Treatment Preferences

This part identifies patients’ preferences and physicians’ perceptions of patients’ preferred treatment strategy in end-of-life care. The content of treatment alternatives included life-prolonging treatment or supportive care.

### Statistical Analysis

Data management and statistical analysis were performed using SPSS 11.0 statistical software (SPSS Inc., Chicago, IL). A frequency distribution was used to describe the demographic data and the distribution of each variable. Mean values and standard deviations were used to analyze the degree of each variable in the beliefs about palliative care and subjective norms. Statistical differences between physicians and patients on attitudes toward truth telling and preferences for treatment strategies were tested using Fisher exact test. A *P* value of <0.05 was considered statistically significant.

## RESULTS

The questionnaire was mailed to 200 physicians in 150 CMTs initially and 177 (88.5%) responded. Of the physicians who returned the questionnaire with participation consent, 2 of their contracted patients were sent the survey questionnaire. After deducting incomplete patient questionnaires, 314 (88.7%) were included in the final analysis.

The respondents in the physician group were mostly male (92.1%) with a mean age of 53.2 (standard deviation [SD] = 9.2). Patient respondents were mostly female (83.1%) with a mean age of 41.3 (SD = 12.2). Among the physicians, 60.5% had experiences of caring for terminal patients, but only 12.4% of them had provided hospice care (Table 1).

**TABLE 1:**
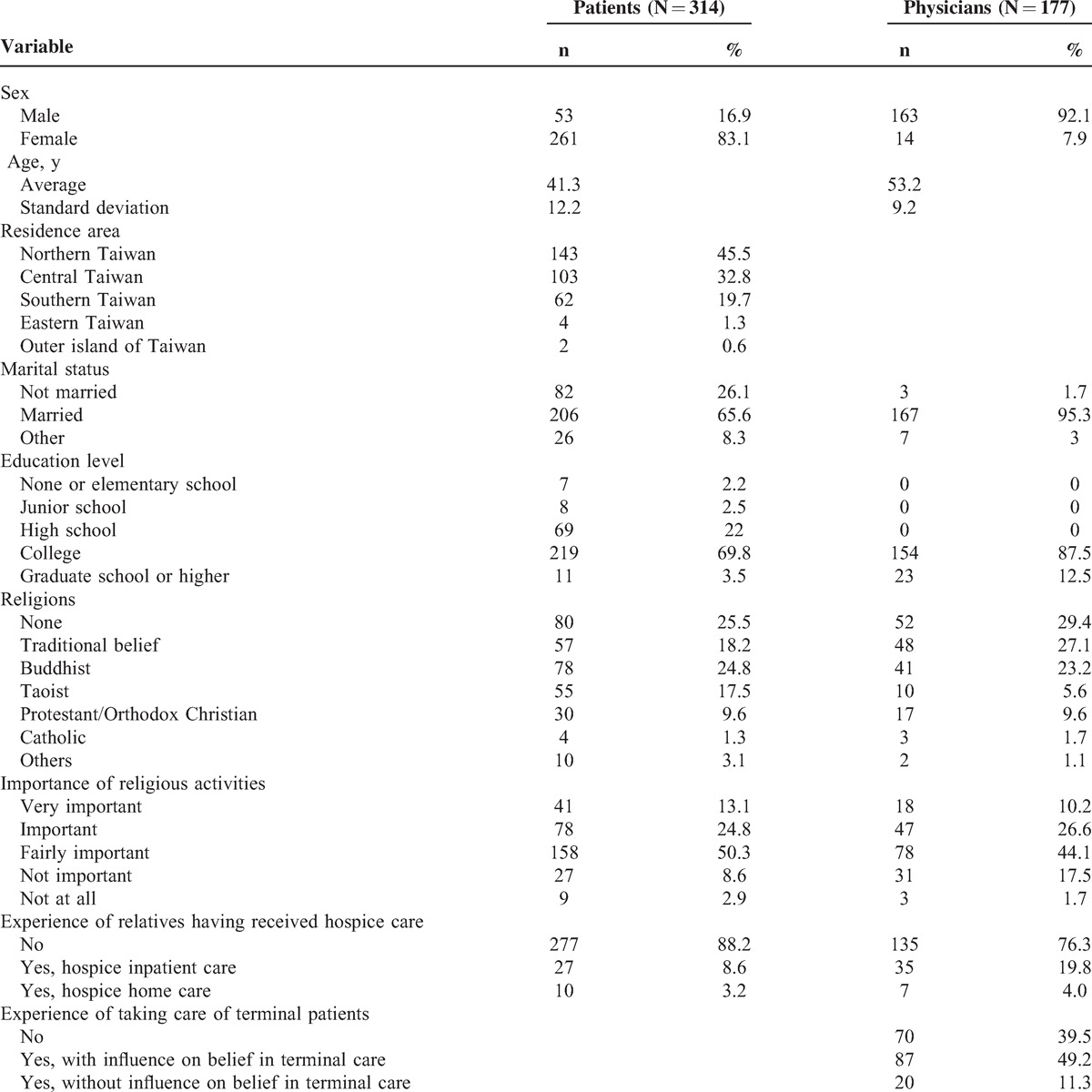
Demographic Characteristics of Participants

### Beliefs and Subjective Norms of the Patients and Physicians

Table 2 indicates possible factors influencing truth telling and treatment strategy preferences among patients and physicians. When considering beliefs, reverse scoring was used in the disadvantages section. A lower score indicated negative beliefs toward hospice care—in other words, perceiving more threats or barriers and fewer benefits. The mean scores of beliefs toward hospice care were 3.60 ± 0.82 in the patient group and 3.66 ± 0.73 in the physician group, which means that both the groups have positive attitudes toward hospice care. Among the 3 subconcepts of beliefs, “benefits” of providing hospice care ranked first in both the groups (4.15 ± 0.22 in patient group, 4.16 ± 0.22 in physician group). In the physician group, the lower-scoring items in perceiving the threats about hospice care were the following: “cure is hopeless for advanced cancer patients” (2.2), “makes me think about death” (2.25), and “unable to easily face dying process and distress” (2.3). The main barriers to hospice care perceived by physicians were the following: “the suffering of facing death” (2.55), “makes patients feel hopeless” (3.21), and “patients feel abandoned” (3.81).

**TABLE 2:**
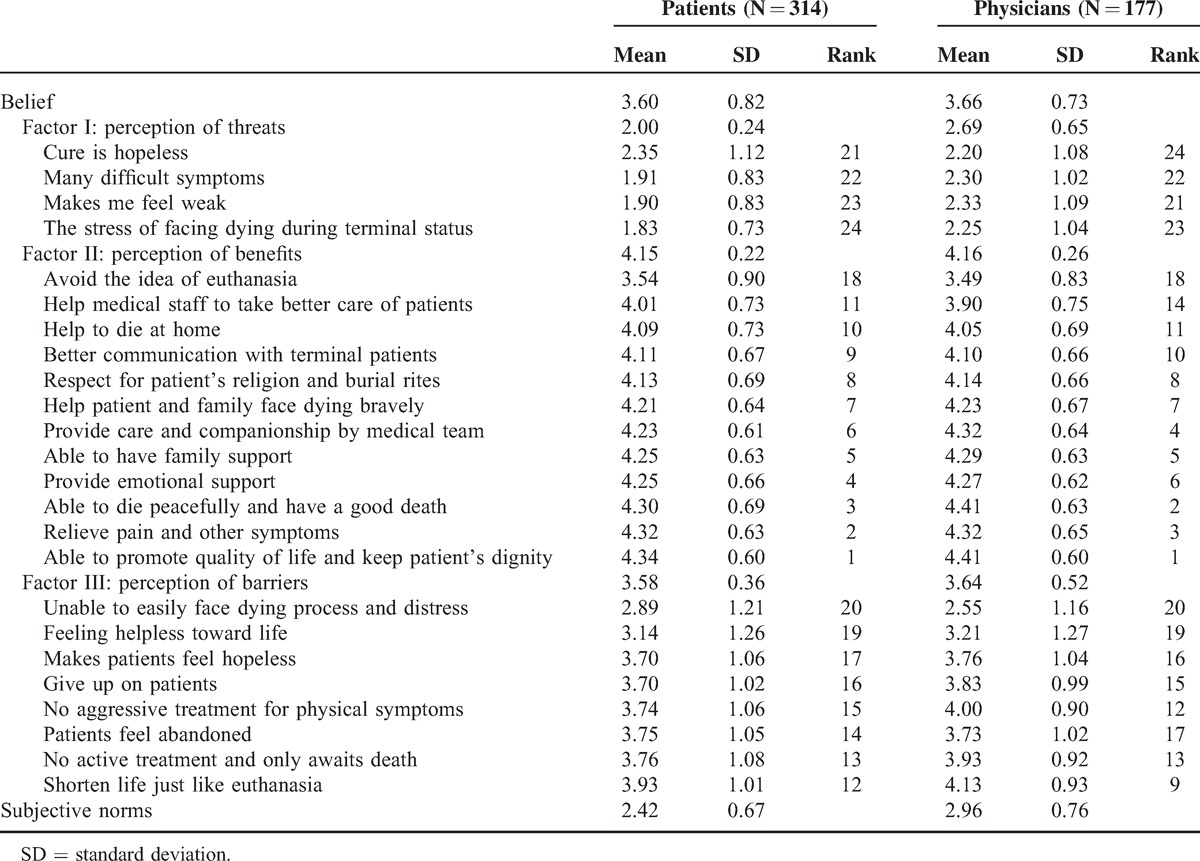
Beliefs of Patients Regarding End-of-Life Care

### Truth Telling

Table 3 demonstrates the survey results for attitudes toward truth telling. Among patients, 94.3% prefer to be informed of any disease prognosis and 5.7% of patients want to understand only curable disease conditions. However, among physicians, 20.9% considered that their patients only wanted to know about curable disease status. If facing cancer, only 1 patient (0.3%) would leave the diagnosis to family and 4 patients (1.3%) did not want to know at all. Only 13.6% of physicians had the impression that their patients preferred that their families understand the cancer diagnosis and 1.7% of physicians believed that patients did not want to know. When encountering terminal disease status, 14.7% of physicians believed that the patients just wanted their families to be informed or they do not want to know at all. However, up to 98.1% of the patients surveyed had the attitude that they wanted to be informed even under terminal conditions.

**TABLE 3:**
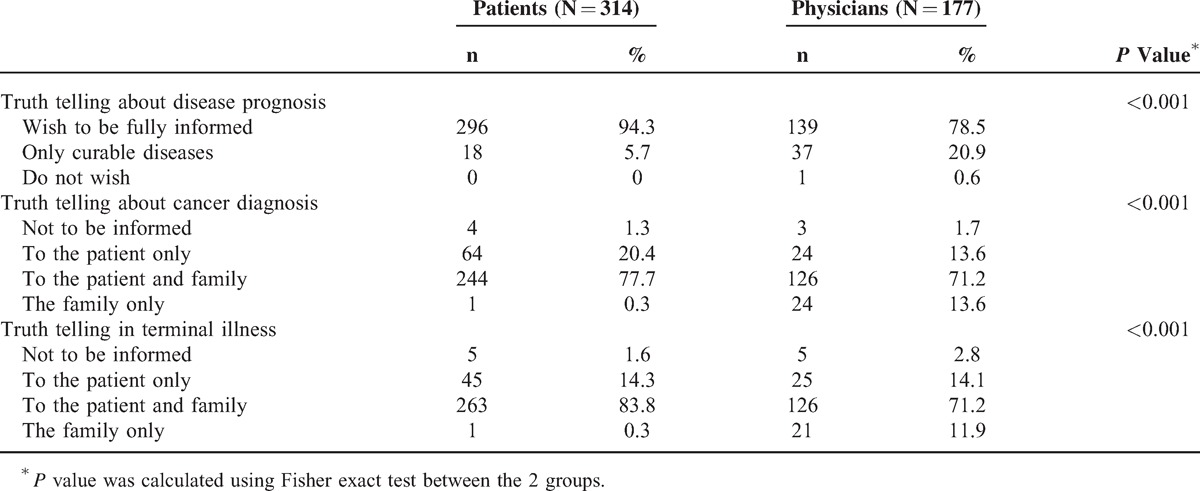
Attitudes Toward Truth Telling

### End-of-Life Treatment Preferences

Facing end of life, nearly 90% of patients strongly or somewhat favored the treatment strategy of supportive care. However, only 15.8% their physicians believed that their patients had this kind of preference. Also, almost half of physicians thought that patients had no preference for treatment strategy, and around one third of surveyed physicians had the perception that patients favored life-prolonging treatment (Table 4).

**TABLE 4:**
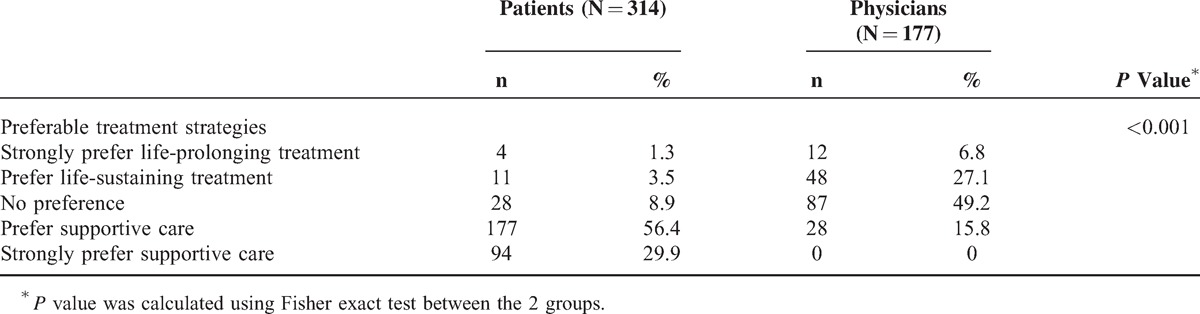
Preferences of Treatment Strategies in Cancer Care Facing End of Life

## DISCUSSION

The present study of the nationwide survey of patients’ preferences versus ACOs’ physicians’ perceptions on truth telling and treatment strategies is especially essential when Taiwan is the first Asian country to enact the Natural Death Act and the National Health Insurance System with the unique physician-led ACO (CMT). The discordance between patients’ preferences and physicians’ perceptions suggests a communication gap and physicians need to make more efforts to communicate with patients during end-of-life decision making. As the nationally representative results suggest, physicians in the ACOs should engage more actively during medical decision making to fully understand patients’ wishes and protect their autonomy for better patient-centered end-of-life care.

Discrepancies between patients’ preferences and physicians’ perceptions about truth telling are demonstrated in this study both in disclosure of cancer diagnosis and terminal conditions. Truth telling ranks first among ethical dilemmas of health professionals in end-of-life care in previous studies,^12,23,24^ and surrogate decision making has many limitations in protecting patients’ best interests such as identification of patients’ values. If physicians with established long-term doctor–patient relationships have the misconception of not disclosing diagnoses to patients, specialists such as oncologists in medical centers with only short-term contact with patients and families may even more easily ignore the need for truth telling. Understanding patients’ preferences for receiving enough relevant medical information under diagnosis of cancer or terminal conditions, physicians should effectively and carefully explain the medical diagnosis to patients, thus giving them sufficient background information for decision making. Also, through the truth telling process, physicians could actively participate in communicating advance care planning and increasing the quality of end-of-life care.

Life-sustaining treatment may become a futile management when a patient faces a terminal condition with poor prognosis. In this circumstance, physicians easily feel frustrated with end-of-life care because curative therapy is the focus of medical education during professional development. If physicians in the ACOs consider that patients’ in terminal care still prefer curative treatments, they may keep referring patients to specialists in order to administer advanced technology treatments or even experimental treatments. This study clearly demonstrates (86.3% of the public surveyed) patients’ need for supportive care, which becomes impossible under the circumstance described earlier. If the results of patient preferences for supportive treatment strategies in end-of-life care can be revealed to physicians, it may effectively reduce physicians’ misunderstanding of treatment goals. Then, community-oriented end-of-life care by physician-led ACOs can be put into practice as patients wish.

Previous surveys in Taiwan have already indicated that providing supportive care to terminal patients is an essential responsibility of continuing care by primary care physicians, demonstrating that 80% to 90% of healthcare professionals are willing to provide hospice care in the community.^25,26^ Moreover, results of those studies showed that the benefits of hospice care received the highest scores from both physicians and patients. Patients’ need for hospice care and physicians’ willingness to provide certain kinds of hospice care are clearly demonstrated. Nevertheless, the present nationwide study showed that 60.5% of community-based physician-led ACOs had the experiences of caring for terminal patients, but only 12.4% of them had provided hospice care to their patients. Providing hospice care training during ACOs’ physicians’ continuing education and overcoming the barriers toward hospice care will enable physicians to be confident in offering patient-centered end-of-life care.

This study has some limitations. First, because Taiwan has its unique National Health Insurance System, the findings here can only be applied to other countries with modification. However, the CMTs functioned as physician-led ACOs in the United States after the enactment of Patient Protection and Affordable Care Act. Moreover, the core value of improving care quality and reducing cost in ACOs is important in every country. In addition, the respondents to the questionnaires may be those who are interested in palliative care, and certain selection bias may exist in the study design. Third, only 2 contracted patients of each physician were selected, which may limit generalizability of results. However, only loyal patients who frequently visited the physicians were included, which means that the physicians surveyed should understand these patients’ preferences well.

In conclusion, significant discrepancies exist between patients’ preferences and physicians’ perceptions toward truth telling and treatment strategies in end-of-life care. The results of this study indicate that it is important to enhance physician–patient communication about end-of-life care preferences in order to achieve the goal of ACOs. Continuing education on communication about end-of-life care during physicians’ professional development would be helpful in the reform strategies of establishing accountable care around the world.
